# UniqTag: Content-Derived Unique and Stable Identifiers for Gene Annotation

**DOI:** 10.1371/journal.pone.0128026

**Published:** 2015-05-28

**Authors:** Shaun D. Jackman, Joerg Bohlmann, İnanç Birol

**Affiliations:** 1 Genome Sciences Centre, British Columbia Cancer Agency, Vancouver, BC, Canada; 2 Graduate Program in Bioinformatics, University of British Columbia, Vancouver, BC, Canada; 3 Michael Smith Laboratories, University of British Columbia, Vancouver, BC, Canada; 4 Department of Medical Genetics, University of British Columbia, Vancouver, BC, Canada; Hellas, GREECE

## Abstract

When working on an ongoing genome sequencing and assembly project, it is rather inconvenient when gene identifiers change from one build of the assembly to the next. The gene labelling system described here, UniqTag, addresses this common challenge. UniqTag assigns a unique identifier to each gene that is a representative *k*-mer, a string of length *k*, selected from the sequence of that gene. Unlike serial numbers, these identifiers are stable between different assemblies and annotations of the same data without requiring that previous annotations be lifted over by sequence alignment. We assign UniqTag identifiers to ten builds of the Ensembl human genome spanning eight years to demonstrate this stability. The implementation of UniqTag in Ruby and an R package are available at https://github.com/sjackman/uniqtag sjackman/uniqtag. The R package is also available from CRAN: install.packages ("uniqtag"). Supplementary material and code to reproduce it is available at https://github.com/sjackman/uniqtag-paper.

## Introduction

The task of annotating the genes of a genome sequence often follows genome assembly. These annotated genes are assigned unique identifiers by which they can be referenced. Assembly and annotation is frequently an iterative process, by refining the method or by the addition of more sequencing data. These gene identifiers would ideally be stable from one assembly and annotation to the next. The common practice is to use serial numbers to identify genes that are annotated by software such as MAKER [1], which, although certainly unique, are not stable between assemblies. A single change in the assembly can result in a total renumbering of the annotated genes.

One solution to stabilize identifiers is to assign them based on the content of the gene sequence. A cryptographic hash function such as SHA (Secure Hash Algorithm) [2] derives a message digest from the sequence, such that two sequences with the same content will have the same message digest, and two sequences that differ will have different message digests. If a cryptographic hash were used to identify a gene, the same gene in two assemblies with identical content would be assigned identical identifiers. Yet, by design a slight change in the sequence, such as a single-character substitution, would result in a completely different digest.

Locality-sensitive hashing in contrast aims to assign items that are similar to the same hash value. A hash function that assigns an identical identifier to a sequence after a modification of that sequence is desirable for labelling the genes of an ongoing genome annotation project. One such locality-sensitive hash function, MinHash, was employed to identify web pages with similar content [3] by selecting a small representative set of words from a web page.

UniqTag is inspired by MinHash. It selects a single representative *k*-mer from a sequence to assign a stable identifier to a gene. These identifiers are intended for systematic labelling of genes rather than assigning biological gene names, as the latter are typically based on biological function or homology to orthologous genes [4]. Assigning UniqTag identifiers to the current assembly requires no knowledge of the previous assemblies.

Annotation of a draft genome that is in progress requires a system by which to identify genes, even if only temporarily and for internal use. Assigning permanent identifiers to a stable genome assembly for public release, such as with complete genome assemblies of model organisms, is a different situation and typically involves a public database of permanently accessioned identifiers, lifting over gene identifiers from one assembly to an updated assembly using sequence alignment, rules of versioning those symbols, and often manual curation. UniqTag on the other hand provides a simple system for quickly assigning stable identifiers with little more effort than assigning serial numbers. Our approach does not require sequence alignment nor in fact any knowledge of previous assemblies to assign identifiers to new assemblies.

## Methods

The UniqTag is defined mathematically as follows. Let Σ be an alphabet, such as the twenty standard amino acids or the four nucleotides. Let Σ^*k*^ be the set of all strings over Σ of length *k*. Let *s* be a string over Σ, such as the peptide or nucleotide sequence of a gene. Let *C*(*s*) be the set of all substrings of *s*, and *C*_*k*_(*s*) be the set of all *k*-mers of *s*, that is, all substrings of *s* with length *k*.

$$\begin{array}{c} C_{k}\left(s\right)=C\left(s\right)\cap \Sigma^{k} \end{array}$$

Let *S* be a set of *n* strings {*s*_0_, …, *s*_*n*_}, such as the peptide or nucleotide sequences of all the annotated genes of a genome assembly. Let *f*(*t*, *S*) be the frequency in *S* of a *k*-mer *t*, defined as the number of strings in *S* that contain the *k*-mer *t*.

$$\begin{array}{c} f\left(t,S\right)=|\{s|t\in C_{k}\left(s\right)\wedge s\in S\}| \end{array}$$

Let the function min*T* define the lexicographically minimal string of a set of strings *T*. That is, if the strings of the set *T* were sorted alphabetically, min*T* would refer to the first string in the list.

Finally, *u*_*k*_(*s*, *S*) is the UniqTag, the lexicographically minimal *k*-mer of those *k*-mers of *s* that are least frequent in *S*.

$$\begin{array}{c} u_{k}\left(s,S\right)=\min\underset{t\in C_{k}\left(s\right)}{\arg\;\min}f\left(t,S\right) \end{array}$$

Typically, *u*_*k*_(*s*, *S*) is the first *k*-mer in an alphabetically sorted list of the *k*-mers of a gene that are unique to that gene.

A UniqTag can be generated from the nucleotide sequence of a gene or the translated peptide sequence of a protein-coding gene. Using the peptide sequence results in a UniqTag that is stable across synonymous changes to the coding sequence as well as to changes in the untranslated regions and introns of the gene. Since the amino acid alphabet is larger than the nucleotide alphabet, fewer characters are required for a *k*-mer to be likely unique, resulting in an aesthetically pleasing shorter identifier.

When two gene models have identical *k*-mer compositions, they would be assigned the same UniqTag. It is also possible that two genes that have no unique *k*-mer and similar *k*-mer composition are assigned the same UniqTag. In such cases, genes that have the same UniqTag are distinguished by adding a numerical suffix to the UniqTag. A UniqTag is formatted as a *k*-mer followed by a hyphen and a number, such as *ARNDCEQGH-1*. For consistency of formatting the suffix is always included, even when the *k*-mer is unique and the numerical suffix is -*1*.

Some genes, such as those found in transposable elements, may occur hundreds of times with little variation. When the repetitive elements are not perfectly identical, the *k*-mers that are unique to each instance of the repetitive element will be chosen preferentially as their UniqTags. Genome sequence assembly may smooth out small variants by collapsing repeats during the initial assembly and then expanding repeats using paired-end reads, after which there may be many identical copies of the repetitive element with no distinguishing unique *k*-mer. Identifying and masking repetitive elements prior to gene annotation will avoid assigning gene identifiers to genes found in repeat elements. Alternatively if assigning gene identifiers to genes found in repeat elements is desirable, a unique *k*-mer could be selected from flanking DNA sequence when the coding sequence is found to be too repetitive. Although this feature is not integrated into our software, this approach can be implemented by assigning UniqTags to both the translated coding sequence and the flanking DNA sequence and selecting the DNA UniqTag when the coding UniqTag is found to be repetitive.

The UniqTag is designed to be stable but will change in the following conditions: (1) when the sequence at the locus of the UniqTag changes; (2) when a least-frequent *k*-mer that is lexicographically smaller than the previous UniqTag is created; (3) when a duplicate *k*-mer is created elsewhere that results in the previous UniqTag no longer being a least-frequent *k*-mer.

UniqTag identifiers are stable to small changes to the gene sequence, such as the correction of small misassemblies, but large changes to the gene sequence or gene model, such as the addition or removal of entire exons, will often result in a change of the UniqTag identifier of that gene. The larger the change of the sequence or gene model, the more likely it is that the UniqTag will change.

The special cases of merging and splitting gene models are interesting. Concatenating two gene models results in a gene whose UniqTag is the minimum of the two previous UniqTags, unless the new UniqTag spans the junction of the two sequences. Similarly when a gene model is split in two, one gene is assigned a new UniqTag and the other retains the previous UniqTag, unless the previous UniqTag spanned the junction.

Importantly and in contrast, unlike naming the genes after the genomic contigs or scaffolds in which they are found, changing the names or the order of the sequences in a genome assembly has no effect on the UniqTag *k*-mers of those genes.

## Results

**Fig 1:**
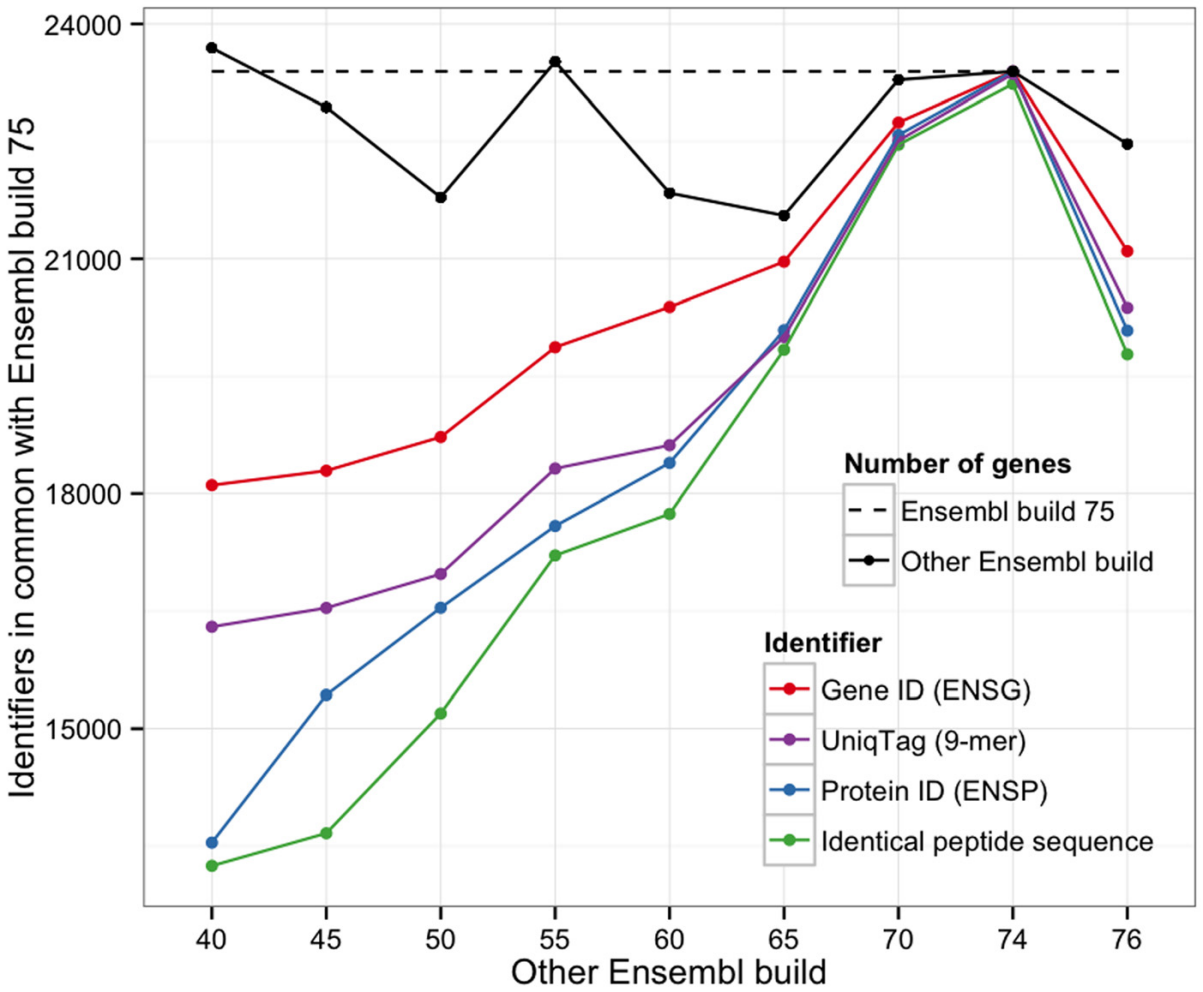
The number of common UniqTag identifiers between build 75 of the Ensembl human genome and nine other builds, the number of common gene and protein identifiers between builds, and the number of genes with peptide sequences that are identical between builds.

To demonstrate the stability and utility of UniqTag, we assigned identifiers to the genes of ten builds of the Ensembl human genome [5] (every fifth build from 40 through 70, and builds 74, 75 and 76) spanning eight years and three major genome assemblies (NCBI36 up to build 54, GRCh37 up to build 75, and GRCh38 for build 76). Ensembl build 75, the final build to use GRCh37, is used as the reference to which all other builds are compared. The number of common UniqTag identifiers between build 75 and nine other builds is shown in Fig 1. A UniqTag identifier of nine amino acids (*k* = 9) was assigned to the first protein sequence, that with the smallest Ensembl protein (ENSP) accession number, of each gene. Also shown is the number of common gene and protein identifiers (ENSG and ENSP accession numbers) between builds and the number of genes with peptide sequences that are identical between builds. Although less stable than the gene ID, the UniqTag is more stable than the protein ID and the peptide sequence. The last build of GRCh37, build 75, and the first build of GRCh38, build 76, for example have 20,376 (90.7%) UniqTag in common and 21,097 (93.9%) ENSG accession numbers in common of the 22,469 genes of build 76.

**Fig 2:**
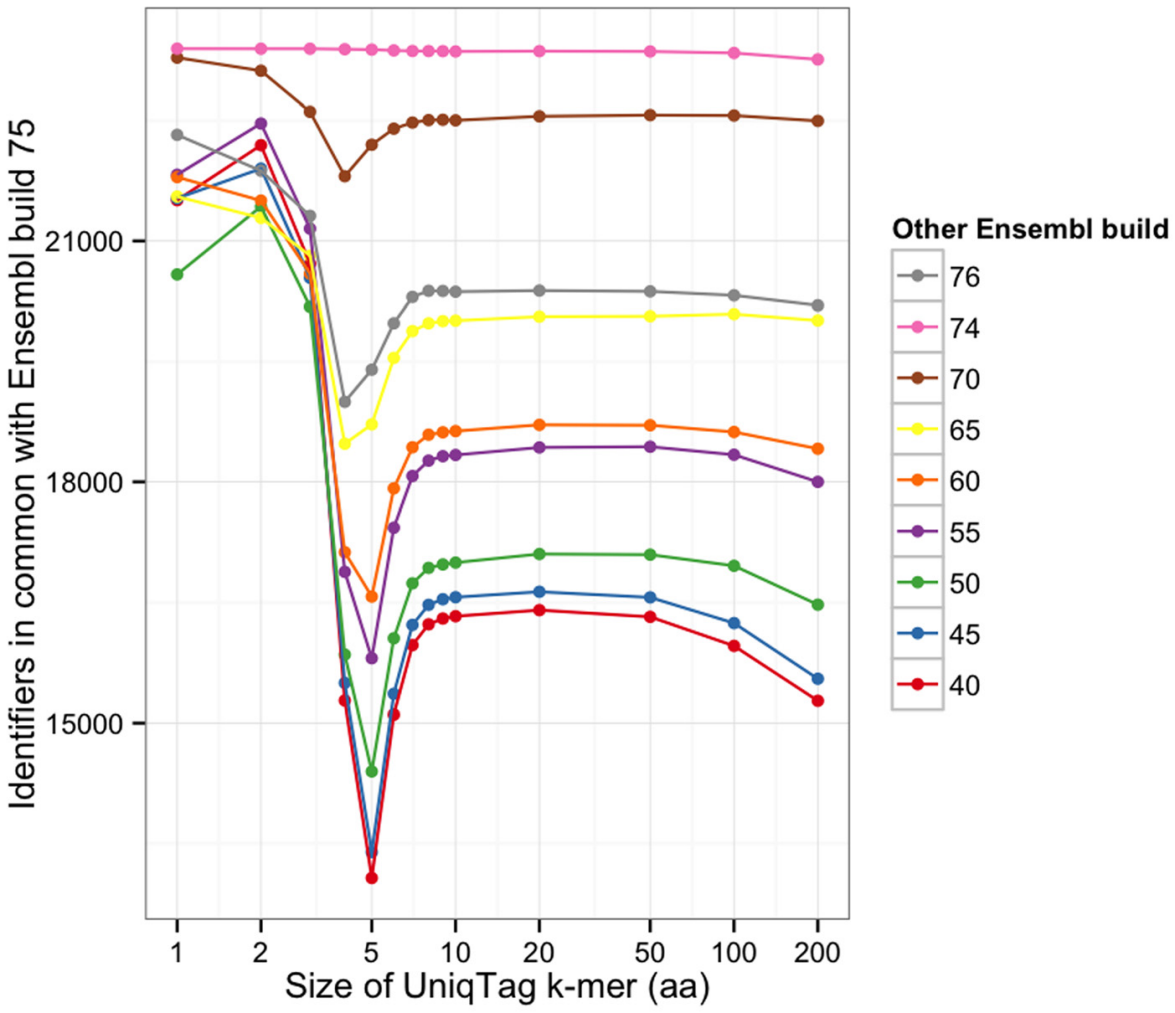
The number of common UniqTag identifiers between build 75 of the Ensembl human genome and nine other builds for different values of *k*.

The stability of the UniqTag is insensitive to the size of the UniqTag identifier for values of *k* between 8 and 50 amino acids, shown in Fig 2. The data for both figures are shown in Table A in S1 File.

As described above, genes with the same peptide sequence result in hash collisions and are disambiguated using a numerical suffix. Duplicate UniqTag *k*-mers due to hash collisions are rare, but can occur in sequences that have no unique *k*-mer, which is most likely with short sequences. NCBI GRCh37 build 75 has 23,393 annotated genes, which have 21,783 (93.1%) distinct peptide sequences. Of these 21,783 distinct sequences, there are 54 (0.25%) UniqTag collisions.

## Conclusions

Whereas the gene and protein identifiers can, with effort, be lifted over from older builds to the newest build, the UniqTag identifier can be generated without any knowledge of previous assemblies, making it a much simpler operation. The number of identical peptide sequences between builds shows the stability that would be expected of using a cryptographic hash value of the peptide sequence as the identifier. The stability of the UniqTag is insensitive to the size of the UniqTag *k*-mer for a large range of *k*.

## Supporting Information

S1 File The data used to plot Figs. 1 and 2, also available in tab-separated values (TSV) format (**Table A**). The source of UniqTag 1.0, implemented in Ruby (**Listing A**). The Makefile script that calculates the data used to plot Figs. 1 and 2 (**Listing B**).
(PDF)
