# Supplementary material for: UniqTag: Content-Derived Unique and Stable Identifiers for Gene Annotation
Source: PLoS One. 2015 May 28;10(5):e0128026. doi: 10.1371/journal.pone.0128026 (PMC4447347; doi:10.1371/journal.pone.0128026)
Supplement: S1 File — The data used to plot Figs. 1 and 2, also available in tab-separated values (TSV) format (Table A). The source of UniqTag 1.0, implemented in Ruby (Listing A). The Makefile script that calculates the data used to plot Figs. 1 and 2 (Listing B). (PDF) [file pone.0128026.s001.pdf]

Supplementary material for UniqTag: Content-derived unique and stable identifiers for gene annotation

Shaun D. Jackman

Supplementary material

The following supplementary material of the UniqTag paper present the code, shown in Listings A and B, and the data, shown in supplementary Table A, used to generate Figures 1 and 2 of the main manuscript.

Load libraries

```
library(ggplot2)
library(knitr) # for kable
library(reshape2)
library(scales) # for alpha
```

Read the data

```
data.orig <- read.delim('UniqTag-supp.tsv',
    colClasses = c(A = 'factor', B = 'factor'))
x <- do.call(rbind, strsplit(as.character(data.orig$Table), '.', fixed = TRUE))
colnames(x) <- c('Data', 'Transform', 'Identifier')
data <- cbind(data.orig, x)
rm(x)
data$k <- as.integer(gsub('^[a-z]*', '', data$Identifier))

build.wide <- with(data,
    data.frame(Build.A = A, Build.B = B,
        Num.A = Only.A + Both, Num.B = Only.B + Both))
build.tall <- melt(build.wide, id.vars = c('Build.A', 'Build.B'),
    variable.name = 'Build', value.name = 'Count')
```

Figure 1. Plot the number of common identifiers vs. other build

The number of common UniqTag identifiers between build 75 of the Ensembl human genome and nine other builds, the number of common gene and protein identifiers between builds, and the number of genes with peptide sequences that are identical between builds.

```
data.subset <- subset(data, data$k == 9 | is.na(data$k))
aes.data <- aes(x = A, y = Both,
    group = Table, colour = Identifier)
aes.build <- aes(x = Build.A, y = Count,
```

```r
    group = Build, linetype = Build, shape = Build)
ggplot() +
    geom_point(aes.data, data.subset) +
    geom_line(aes.data, data.subset) +
    scale_colour_brewer(palette = 'Set1',
        breaks = c('gene', 'uniqtag9', 'id', 'seq'),
        labels = c('Gene ID (ENSG)', 'UniqTag (9-mer)',
            'Protein ID (ENSP)', 'Identical peptide sequence')) +

    geom_point(aes.build, build.tall) +
    geom_line(aes.build, build.tall) +
    scale_linetype_manual(name = 'Number of genes',
        breaks = c('Num.B', 'Num.A'),
        labels = c('Ensembl build 75', 'Other Ensembl build'),
        values = c('solid', 'dashed')) +
    scale_shape_manual(name = 'Number of genes',
        breaks = c('Num.B', 'Num.A'),
        labels = c('Ensembl build 75', 'Other Ensembl build'),
        values = c(20, 32)) +

    theme_bw() +
    theme(legend.position = c(1.0, 0),
        legend.justification = c(1, 0),
        legend.box.just = 'right',
        legend.background = element_rect(fill = alpha('white', 0))) +
    xlab('Other Ensembl build') +
    ylab('Identifiers in common with Ensembl build 75')
```

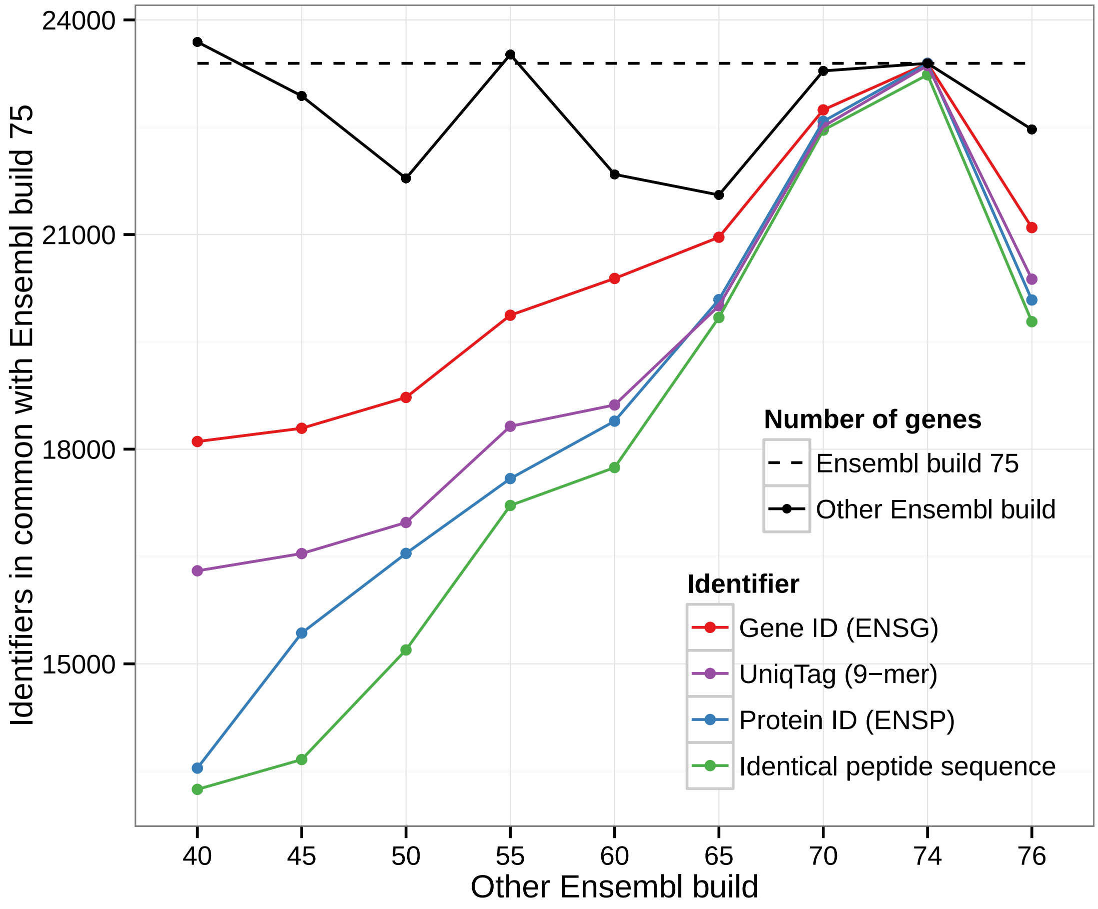

Figure 2. Plot the number of common identifiers vs. *k*

The number of common UniqTag identifiers between build 75 of the Ensembl human genome and nine other builds for different values of k.

```
ggplot(na.omit(data), aes(x = k, y = Both, group = A, colour = A)) +
    geom_point() +
    geom_line() +
    scale_x_continuous(trans = log_trans(),
        breaks = c(1, 2, 5, 10, 20, 50, 100, 200)) +
    scale_colour_brewer(name = 'Other Ensembl build', palette = 'Set1') +
    guides(colour = guide_legend(reverse = TRUE)) +
    theme_bw() +
    xlab('Size of UniqTag k-mer (aa)') +
    ylab('Identifiers in common with Ensembl build 75')
```

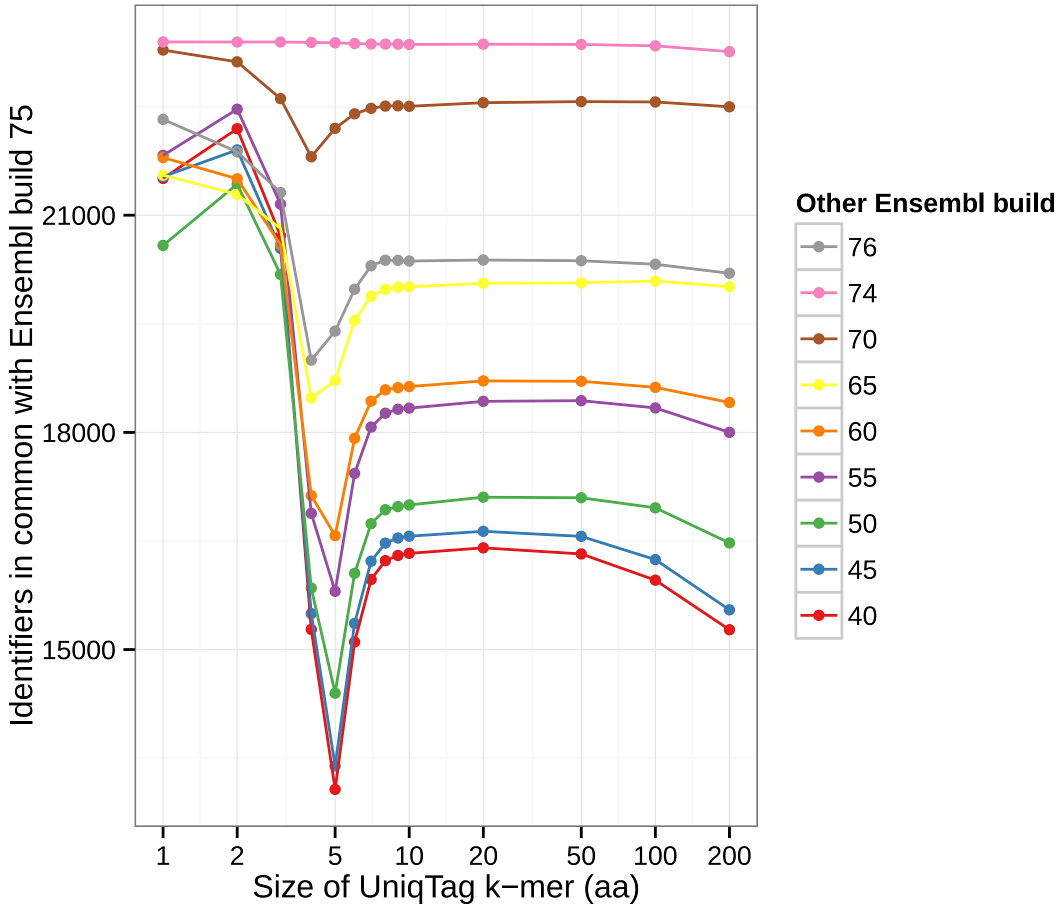

Listing A. UniqTag 1.0

This listing shows the source of UniqTag 1.0, implemented in Ruby.

```ruby
#!/usr/bin/env ruby
# Determine a unique substring (k-mer) of each string
# Copyright 2014 Shaun Jackman

require 'optparse'

class String
  # Iterate over each k-mer
  def each_kmer k
    return enum_for(:each_kmer, k) unless block_given?
    (0 .. length - k).each { |i|
      kmer = self[i, k]
      yield kmer unless kmer =~ /~/
    }
  end
end

class Array
  # Append a serial number to distinguish duplicate strings
```

```ruby
  def dedup
    each_with_object(Hash.new(0)).map { |x, count|
      "#{x}-#{count[x] += 1}"
    }
  end
end

# Count the k-mers in a set of strings
def count_kmer seqs, k
  seqs.each_with_object(Hash.new(0)) { |seq, counts|
    seq.each_kmer(k).to_a.uniq.each { |kmer|
      counts[kmer] += 1
    }
  }
end

# Return the unique tag of the specified string
def get_tag seq, kmer_counts, k
  _, tag = seq.each_kmer(k).map { |kmer|
    [kmer_counts[kmer], kmer]
  }.min
  tag || seq.split('~').min
end

# Parse command line options
k = 9
OptionParser.new do |opts|
  opts.banner = "Usage: uniqtag [-k N] [FILE]..."
  opts.version = "0.1.0"
  opts.release = nil

  opts.on("-k", "--kmer N", OptionParser::DecimalInteger,
      "Size of the unique tag (default 9)") do |n|
    k = n
  end
end.parse!

# Read strings and write unique tags
seqs = ARGF.each_line.reject { |s|
  s =~ /^>/
}.map { |s|
  s.chomp.upcase
}
kmer_counts = count_kmer seqs, k
puts seqs.map { |seq| get_tag(seq, kmer_counts, k) }.dedup
```

Listing B. Calculate the number of common identifiers

This Makefile script calculates the data used to plot the above figures.

```
# The supplementary material for the UniqTag paper
# UniqTag: Content-derived unique and stable identifiers for gene annotation
# Copyright 2014 Shaun Jackman

# Download the data and compute the results
all: UniqTag.tsv

# Remove all computed files
clean:
	rm -f *.comm *.gene *.id *.seq *.sort *.tsv *.uniqtag *.venn

# Install dependencies
install-deps: /usr/local/bin/brew
	brew install coreutils seqtk uniqtag wget

# Check for Homebrew
/usr/local/bin/brew:
	@if brew --version >/dev/null 2>/dev/null; then \
		echo Install Homebrew http://brew.sh/ or Linuxbrew http://brew.sh/linuxbrew/; \
	fi

.PHONY: all clean install-deps
.DELETE_ON_ERROR:
.SECONDARY:

# Download Ensembl Human genome NCBI36 build 40
Homo_sapiens.NCBI36.40.pep.all.fa.gz:
	wget ftp://ftp.ensembl.org/pub/release-40/homo_sapiens_40_36b/data/fasta/pep/Homo_sapiens.NCBI36.40

# Download Ensembl Human genome NCBI36 build 45
Homo_sapiens.NCBI36.45.pep.all.fa.gz:
	wget ftp://ftp.ensembl.org/pub/release-45/homo_sapiens_45_36g/data/fasta/pep/Homo_sapiens.NCBI36.45

# Download Ensembl Human genome NCBI36
Homo_sapiens.NCBI36.%.pep.all.fa.gz:
	wget ftp://ftp.ensembl.org/pub/release-$*/fasta/homo_sapiens/pep/Homo_sapiens.NCBI36.$*.pep.all.fa.

# Download Ensembl Human genome GRCh37
Homo_sapiens.GRCh37.%.pep.all.fa.gz:
	wget ftp://ftp.ensembl.org/pub/release-$*/fasta/homo_sapiens/pep/Homo_sapiens.GRCh37.$*.pep.all.fa.

# Download Ensembl Human genome GRCh38
Homo_sapiens.GRCh38.%.pep.all.fa.gz:
	wget -O $@ ftp://ftp.ensembl.org/pub/release-$*/fasta/homo_sapiens/pep/Homo_sapiens.GRCh38.pep.all.

# Uncompress FASTA and remove line breaks
%.fa: %.fa.gz
	seqtk seq $< >$@

# Remove the headers from a FASTA file
%.seq: %.fa
	grep -v '^>' $< >$@
```

```
# Convert a FASTA file to sorted TSV of ID, gene name and sequence
%.all.fa.tsv: %.all.fa
	awk -vORS='' '{print $$1 "\t" $$4; getline; print "\t" $$0 "\n" }' $< |sort -k2,2 -k1 >$@

# Keep the first protein isoform in the FASTA file
%.uniqgene.fa: %.fa.tsv
	awk 'x[$$2]++ == 0 { print $$1 " " $$2 "\n" $$3 }' $< >$@

# Join all protein isoforms separated by tilde
%.allgene.fa: %.fa.tsv
	awk 'x[$$2]++ == 0 { print $$1 " " $$2 "\n" $$3; next } \
		{ print "~" $$3 }' $< |seqtk seq - >$@

# Extract the gene name from the FASTA header
%.gene: %.fa
	sed -En 's/^>.*gene:([^ ]*).*/\1/p' $< >$@

# Extract the ID from the FASTA header
%.id: %.fa
	sed -En 's/^>([^ ]*).*/\1/p' $< >$@

# Compute the UniqTag for each sequence in the FASTA file
ks=1 2 3 4 5 6 7 8 9 10 20 50 100 200
$(foreach k, $(ks), $(eval %.uniqtag$k: %.fa; uniqtag -k$k $$< >$$@))

# Join the gene name, ID and UniqTag into a TSV file
%.tsv: %.gene %.id %.uniqtag7
	(printf "gene\tid\tuniqtag\n" && paste $^) >$@

# Join the TSV of identifiers of two builds on the gene name
Homo_sapiens.GRCh37.70.75.%.tsv: Homo_sapiens.GRCh37.70.%.tsv Homo_sapiens.GRCh37.75.%.tsv
	join $^ |tr ' ' '\t' >$@

# Sort the file
%.sort: %
	sort $< >$@

# Compare an older Ensembl build to build 75
# Note: BSD comm has a bug possibly related to long lines and so GNU comm is
# used instead.
Homo_sapiens.Ensembl.40.75.%.comm: Homo_sapiens.NCBI36.40.%.sort Homo_sapiens.GRCh37.75.%.sort
	gcomm $^ >$@

Homo_sapiens.Ensembl.45.75.%.comm: Homo_sapiens.NCBI36.45.%.sort Homo_sapiens.GRCh37.75.%.sort
	gcomm $^ >$@

Homo_sapiens.Ensembl.50.75.%.comm: Homo_sapiens.NCBI36.50.%.sort Homo_sapiens.GRCh37.75.%.sort
	gcomm $^ >$@

Homo_sapiens.GRCh37.55.75.%.comm: Homo_sapiens.GRCh37.55.%.sort Homo_sapiens.GRCh37.75.%.sort
	gcomm $^ >$@

Homo_sapiens.GRCh37.60.75.%.comm: Homo_sapiens.GRCh37.60.%.sort Homo_sapiens.GRCh37.75.%.sort
```

```
    gcomm $^ >$@

Homo_sapiens.GRCh37.65.75.%.comm: Homo_sapiens.GRCh37.65.%.sort Homo_sapiens.GRCh37.75.%.sort
    gcomm $^ >$@

Homo_sapiens.GRCh37.70.75.%.comm: Homo_sapiens.GRCh37.70.%.sort Homo_sapiens.GRCh37.75.%.sort
    gcomm $^ >$@

Homo_sapiens.GRCh37.74.75.%.comm: Homo_sapiens.GRCh37.74.%.sort Homo_sapiens.GRCh37.75.%.sort
    gcomm $^ >$@

Homo_sapiens.GRCh38.76.75.%.comm: Homo_sapiens.GRCh38.76.%.sort Homo_sapiens.GRCh37.75.%.sort
    gcomm $^ >$@

# Count the overlap of two sets
%.venn: %.comm
    printf "%u\t%u\t%u\n" `grep -c $$'^[^\t]' $<` \
        `grep -c $$'^\t\t' $<` \
        `grep -c $$'^\t[^\t]' $<` >$@

# Create the experimental design table
%-design.tsv:
    printf "%s\t%s\t%s\n" >$@ \
        Table A B \
        $* 40 75 \
        $* 45 75 \
        $* 50 75 \
        $* 55 75 \
        $* 60 75 \
        $* 65 75 \
        $* 70 75 \
        $* 74 75 \
        $* 76 75

# Compute the experimental data table
%-data.tsv: \
        Homo_sapiens.Ensembl.40.75.pep.%.venn \
        Homo_sapiens.Ensembl.45.75.pep.%.venn \
        Homo_sapiens.Ensembl.50.75.pep.%.venn \
        Homo_sapiens.GRCh37.55.75.pep.%.venn \
        Homo_sapiens.GRCh37.60.75.pep.%.venn \
        Homo_sapiens.GRCh37.65.75.pep.%.venn \
        Homo_sapiens.GRCh37.70.75.pep.%.venn \
        Homo_sapiens.GRCh37.74.75.pep.%.venn \
        Homo_sapiens.GRCh38.76.75.pep.%.venn
    (printf 'Only.A\tBoth\tOnly.B\n' && cat $^) >$@

# Join the experimental design and data tables
%.tsv: %-design.tsv %-data.tsv
    paste $^ >$@

# Compute the data table
UniqTag.tsv: \
```

```
        all.uniqgene.gene.tsv \
        all.uniqgene.id.tsv \
        all.uniqgene.seq.tsv \
        all.uniqgene.uniqtag1.tsv \
        all.uniqgene.uniqtag2.tsv \
        all.uniqgene.uniqtag3.tsv \
        all.uniqgene.uniqtag4.tsv \
        all.uniqgene.uniqtag5.tsv \
        all.uniqgene.uniqtag6.tsv \
        all.uniqgene.uniqtag7.tsv \
        all.uniqgene.uniqtag8.tsv \
        all.uniqgene.uniqtag9.tsv \
        all.uniqgene.uniqtag10.tsv \
        all.uniqgene.uniqtag20.tsv \
        all.uniqgene.uniqtag50.tsv \
        all.uniqgene.uniqtag100.tsv \
        all.uniqgene.uniqtag200.tsv
    (head -n1 $< && tail -qn+2 $^) >$@
```

Table A. The number of common identifiers

These data are used to plot the above figures. They are also available in tab-separated values (TSV) format.

```
kable(data)
```

Table	A	B	Only.A	Both	Only.B	Data	Transform	Identifier	k
all.uniqgene.gene	40	75	5585	18107	5286	all	uniqgene	gene	NA
all.uniqgene.gene	45	75	4645	18292	5101	all	uniqgene	gene	NA
all.uniqgene.gene	50	75	3062	18723	4670	all	uniqgene	gene	NA
all.uniqgene.gene	55	75	3644	19872	3521	all	uniqgene	gene	NA
all.uniqgene.gene	60	75	1455	20386	3007	all	uniqgene	gene	NA
all.uniqgene.gene	65	75	591	20962	2431	all	uniqgene	gene	NA
all.uniqgene.gene	70	75	545	22742	651	all	uniqgene	gene	NA
all.uniqgene.gene	74	75	0	23393	0	all	uniqgene	gene	NA
all.uniqgene.gene	76	75	1372	21097	2296	all	uniqgene	gene	NA
all.uniqgene.id	40	75	10150	13542	9851	all	uniqgene	id	NA
all.uniqgene.id	45	75	7507	15430	7963	all	uniqgene	id	NA
all.uniqgene.id	50	75	5242	16543	6850	all	uniqgene	id	NA
all.uniqgene.id	55	75	5927	17589	5804	all	uniqgene	id	NA
all.uniqgene.id	60	75	3449	18392	5001	all	uniqgene	id	NA
all.uniqgene.id	65	75	1463	20090	3303	all	uniqgene	id	NA
all.uniqgene.id	70	75	705	22582	811	all	uniqgene	id	NA
all.uniqgene.id	74	75	0	23393	0	all	uniqgene	id	NA
all.uniqgene.id	76	75	2384	20085	3308	all	uniqgene	id	NA
all.uniqgene.seq	40	75	10447	13245	10148	all	uniqgene	seq	NA
all.uniqgene.seq	45	75	9275	13662	9731	all	uniqgene	seq	NA
all.uniqgene.seq	50	75	6591	15194	8199	all	uniqgene	seq	NA
all.uniqgene.seq	55	75	6303	17213	6180	all	uniqgene	seq	NA
all.uniqgene.seq	60	75	4098	17743	5650	all	uniqgene	seq	NA
all.uniqgene.seq	65	75	1713	19840	3553	all	uniqgene	seq	NA
all.uniqgene.seq	70	75	828	22459	934	all	uniqgene	seq	NA

Table	A	B	Only.A	Both	Only.B	Data	Transform	Identifier	k
all.uniqgene.seq	74	75	160	23233	160	all	uniqgene	seq	NA
all.uniqgene.seq	76	75	2687	19782	3611	all	uniqgene	seq	NA
all.uniqgene.uniqtag1	40	75	2184	21508	1885	all	uniqgene	uniqtag1	1
all.uniqgene.uniqtag1	45	75	1405	21532	1861	all	uniqgene	uniqtag1	1
all.uniqgene.uniqtag1	50	75	1203	20582	2811	all	uniqgene	uniqtag1	1
all.uniqgene.uniqtag1	55	75	1690	21826	1567	all	uniqgene	uniqtag1	1
all.uniqgene.uniqtag1	60	75	45	21796	1597	all	uniqgene	uniqtag1	1
all.uniqgene.uniqtag1	65	75	0	21553	1840	all	uniqgene	uniqtag1	1
all.uniqgene.uniqtag1	70	75	6	23281	112	all	uniqgene	uniqtag1	1
all.uniqgene.uniqtag1	74	75	0	23393	0	all	uniqgene	uniqtag1	1
all.uniqgene.uniqtag1	76	75	146	22323	1070	all	uniqgene	uniqtag1	1
all.uniqgene.uniqtag2	40	75	1498	22194	1199	all	uniqgene	uniqtag2	2
all.uniqgene.uniqtag2	45	75	1035	21902	1491	all	uniqgene	uniqtag2	2
all.uniqgene.uniqtag2	50	75	356	21429	1964	all	uniqgene	uniqtag2	2
all.uniqgene.uniqtag2	55	75	1052	22464	929	all	uniqgene	uniqtag2	2
all.uniqgene.uniqtag2	60	75	338	21503	1890	all	uniqgene	uniqtag2	2
all.uniqgene.uniqtag2	65	75	266	21287	2106	all	uniqgene	uniqtag2	2
all.uniqgene.uniqtag2	70	75	169	23118	275	all	uniqgene	uniqtag2	2
all.uniqgene.uniqtag2	74	75	1	23392	1	all	uniqgene	uniqtag2	2
all.uniqgene.uniqtag2	76	75	594	21875	1518	all	uniqgene	uniqtag2	2
all.uniqgene.uniqtag3	40	75	2975	20717	2676	all	uniqgene	uniqtag3	3
all.uniqgene.uniqtag3	45	75	2396	20541	2852	all	uniqgene	uniqtag3	3
all.uniqgene.uniqtag3	50	75	1603	20182	3211	all	uniqgene	uniqtag3	3
all.uniqgene.uniqtag3	55	75	2363	21153	2240	all	uniqgene	uniqtag3	3
all.uniqgene.uniqtag3	60	75	1249	20592	2801	all	uniqgene	uniqtag3	3
all.uniqgene.uniqtag3	65	75	737	20816	2577	all	uniqgene	uniqtag3	3
all.uniqgene.uniqtag3	70	75	677	22610	783	all	uniqgene	uniqtag3	3
all.uniqgene.uniqtag3	74	75	1	23392	1	all	uniqgene	uniqtag3	3
all.uniqgene.uniqtag3	76	75	1156	21313	2080	all	uniqgene	uniqtag3	3
all.uniqgene.uniqtag4	40	75	8414	15278	8115	all	uniqgene	uniqtag4	4
all.uniqgene.uniqtag4	45	75	7440	15497	7896	all	uniqgene	uniqtag4	4
all.uniqgene.uniqtag4	50	75	5935	15850	7543	all	uniqgene	uniqtag4	4
all.uniqgene.uniqtag4	55	75	6634	16882	6511	all	uniqgene	uniqtag4	4
all.uniqgene.uniqtag4	60	75	4714	17127	6266	all	uniqgene	uniqtag4	4
all.uniqgene.uniqtag4	65	75	3078	18475	4918	all	uniqgene	uniqtag4	4
all.uniqgene.uniqtag4	70	75	1480	21807	1586	all	uniqgene	uniqtag4	4
all.uniqgene.uniqtag4	74	75	7	23386	7	all	uniqgene	uniqtag4	4
all.uniqgene.uniqtag4	76	75	3471	18998	4395	all	uniqgene	uniqtag4	4
all.uniqgene.uniqtag5	40	75	10623	13069	10324	all	uniqgene	uniqtag5	5
all.uniqgene.uniqtag5	45	75	9545	13392	10001	all	uniqgene	uniqtag5	5
all.uniqgene.uniqtag5	50	75	7387	14398	8995	all	uniqgene	uniqtag5	5
all.uniqgene.uniqtag5	55	75	7711	15805	7588	all	uniqgene	uniqtag5	5
all.uniqgene.uniqtag5	60	75	5267	16574	6819	all	uniqgene	uniqtag5	5
all.uniqgene.uniqtag5	65	75	2836	18717	4676	all	uniqgene	uniqtag5	5
all.uniqgene.uniqtag5	70	75	1087	22200	1193	all	uniqgene	uniqtag5	5
all.uniqgene.uniqtag5	74	75	12	23381	12	all	uniqgene	uniqtag5	5
all.uniqgene.uniqtag5	76	75	3070	19399	3994	all	uniqgene	uniqtag5	5
all.uniqgene.uniqtag6	40	75	8587	15105	8288	all	uniqgene	uniqtag6	6
all.uniqgene.uniqtag6	45	75	7575	15362	8031	all	uniqgene	uniqtag6	6
all.uniqgene.uniqtag6	50	75	5731	16054	7339	all	uniqgene	uniqtag6	6
all.uniqgene.uniqtag6	55	75	6083	17433	5960	all	uniqgene	uniqtag6	6
all.uniqgene.uniqtag6	60	75	3922	17919	5474	all	uniqgene	uniqtag6	6

Table	A	B	Only.A	Both	Only.B	Data	Transform	Identifier	k
all.uniqgene.uniqtag6	65	75	2007	19546	3847	all	uniqgene	uniqtag6	6
all.uniqgene.uniqtag6	70	75	887	22400	993	all	uniqgene	uniqtag6	6
all.uniqgene.uniqtag6	74	75	22	23371	22	all	uniqgene	uniqtag6	6
all.uniqgene.uniqtag6	76	75	2492	19977	3416	all	uniqgene	uniqtag6	6
all.uniqgene.uniqtag7	40	75	7723	15969	7424	all	uniqgene	uniqtag7	7
all.uniqgene.uniqtag7	45	75	6716	16221	7172	all	uniqgene	uniqtag7	7
all.uniqgene.uniqtag7	50	75	5046	16739	6654	all	uniqgene	uniqtag7	7
all.uniqgene.uniqtag7	55	75	5443	18073	5320	all	uniqgene	uniqtag7	7
all.uniqgene.uniqtag7	60	75	3410	18431	4962	all	uniqgene	uniqtag7	7
all.uniqgene.uniqtag7	65	75	1673	19880	3513	all	uniqgene	uniqtag7	7
all.uniqgene.uniqtag7	70	75	811	22476	917	all	uniqgene	uniqtag7	7
all.uniqgene.uniqtag7	74	75	29	23364	29	all	uniqgene	uniqtag7	7
all.uniqgene.uniqtag7	76	75	2167	20302	3091	all	uniqgene	uniqtag7	7
all.uniqgene.uniqtag8	40	75	7464	16228	7165	all	uniqgene	uniqtag8	8
all.uniqgene.uniqtag8	45	75	6466	16471	6922	all	uniqgene	uniqtag8	8
all.uniqgene.uniqtag8	50	75	4853	16932	6461	all	uniqgene	uniqtag8	8
all.uniqgene.uniqtag8	55	75	5251	18265	5128	all	uniqgene	uniqtag8	8
all.uniqgene.uniqtag8	60	75	3253	18588	4805	all	uniqgene	uniqtag8	8
all.uniqgene.uniqtag8	65	75	1576	19977	3416	all	uniqgene	uniqtag8	8
all.uniqgene.uniqtag8	70	75	780	22507	886	all	uniqgene	uniqtag8	8
all.uniqgene.uniqtag8	74	75	30	23363	30	all	uniqgene	uniqtag8	8
all.uniqgene.uniqtag8	76	75	2091	20378	3015	all	uniqgene	uniqtag8	8
all.uniqgene.uniqtag9	40	75	7392	16300	7093	all	uniqgene	uniqtag9	9
all.uniqgene.uniqtag9	45	75	6396	16541	6852	all	uniqgene	uniqtag9	9
all.uniqgene.uniqtag9	50	75	4810	16975	6418	all	uniqgene	uniqtag9	9
all.uniqgene.uniqtag9	55	75	5196	18320	5073	all	uniqgene	uniqtag9	9
all.uniqgene.uniqtag9	60	75	3223	18618	4775	all	uniqgene	uniqtag9	9
all.uniqgene.uniqtag9	65	75	1549	20004	3389	all	uniqgene	uniqtag9	9
all.uniqgene.uniqtag9	70	75	776	22511	882	all	uniqgene	uniqtag9	9
all.uniqgene.uniqtag9	74	75	31	23362	31	all	uniqgene	uniqtag9	9
all.uniqgene.uniqtag9	76	75	2093	20376	3017	all	uniqgene	uniqtag9	9
all.uniqgene.uniqtag10	40	75	7363	16329	7064	all	uniqgene	uniqtag10	10
all.uniqgene.uniqtag10	45	75	6371	16566	6827	all	uniqgene	uniqtag10	10
all.uniqgene.uniqtag10	50	75	4787	16998	6395	all	uniqgene	uniqtag10	10
all.uniqgene.uniqtag10	55	75	5181	18335	5058	all	uniqgene	uniqtag10	10
all.uniqgene.uniqtag10	60	75	3208	18633	4760	all	uniqgene	uniqtag10	10
all.uniqgene.uniqtag10	65	75	1543	20010	3383	all	uniqgene	uniqtag10	10
all.uniqgene.uniqtag10	70	75	783	22504	889	all	uniqgene	uniqtag10	10
all.uniqgene.uniqtag10	74	75	35	23358	35	all	uniqgene	uniqtag10	10
all.uniqgene.uniqtag10	76	75	2102	20367	3026	all	uniqgene	uniqtag10	10
all.uniqgene.uniqtag20	40	75	7287	16405	6988	all	uniqgene	uniqtag20	20
all.uniqgene.uniqtag20	45	75	6303	16634	6759	all	uniqgene	uniqtag20	20
all.uniqgene.uniqtag20	50	75	4680	17105	6288	all	uniqgene	uniqtag20	20
all.uniqgene.uniqtag20	55	75	5087	18429	4964	all	uniqgene	uniqtag20	20
all.uniqgene.uniqtag20	60	75	3130	18711	4682	all	uniqgene	uniqtag20	20
all.uniqgene.uniqtag20	65	75	1493	20060	3333	all	uniqgene	uniqtag20	20
all.uniqgene.uniqtag20	70	75	733	22554	839	all	uniqgene	uniqtag20	20
all.uniqgene.uniqtag20	74	75	31	23362	31	all	uniqgene	uniqtag20	20
all.uniqgene.uniqtag20	76	75	2088	20381	3012	all	uniqgene	uniqtag20	20
all.uniqgene.uniqtag50	40	75	7371	16321	7072	all	uniqgene	uniqtag50	50
all.uniqgene.uniqtag50	45	75	6373	16564	6829	all	uniqgene	uniqtag50	50
all.uniqgene.uniqtag50	50	75	4688	17097	6296	all	uniqgene	uniqtag50	50

Table	A	B	Only.A	Both	Only.B	Data	Transform	Identifier	k
all.uniqgene.uniqtag50	55	75	5078	18438	4955	all	uniqgene	uniqtag50	50
all.uniqgene.uniqtag50	60	75	3135	18706	4687	all	uniqgene	uniqtag50	50
all.uniqgene.uniqtag50	65	75	1488	20065	3328	all	uniqgene	uniqtag50	50
all.uniqgene.uniqtag50	70	75	718	22569	824	all	uniqgene	uniqtag50	50
all.uniqgene.uniqtag50	74	75	35	23358	35	all	uniqgene	uniqtag50	50
all.uniqgene.uniqtag50	76	75	2098	20371	3022	all	uniqgene	uniqtag50	50
all.uniqgene.uniqtag100	40	75	7733	15959	7434	all	uniqgene	uniqtag100	100
all.uniqgene.uniqtag100	45	75	6694	16243	7150	all	uniqgene	uniqtag100	100
all.uniqgene.uniqtag100	50	75	4827	16958	6435	all	uniqgene	uniqtag100	100
all.uniqgene.uniqtag100	55	75	5178	18338	5055	all	uniqgene	uniqtag100	100
all.uniqgene.uniqtag100	60	75	3219	18622	4771	all	uniqgene	uniqtag100	100
all.uniqgene.uniqtag100	65	75	1462	20091	3302	all	uniqgene	uniqtag100	100
all.uniqgene.uniqtag100	70	75	723	22564	829	all	uniqgene	uniqtag100	100
all.uniqgene.uniqtag100	74	75	54	23339	54	all	uniqgene	uniqtag100	100
all.uniqgene.uniqtag100	76	75	2147	20322	3071	all	uniqgene	uniqtag100	100
all.uniqgene.uniqtag200	40	75	8418	15274	8119	all	uniqgene	uniqtag200	200
all.uniqgene.uniqtag200	45	75	7388	15549	7844	all	uniqgene	uniqtag200	200
all.uniqgene.uniqtag200	50	75	5312	16473	6920	all	uniqgene	uniqtag200	200
all.uniqgene.uniqtag200	55	75	5516	18000	5393	all	uniqgene	uniqtag200	200
all.uniqgene.uniqtag200	60	75	3428	18413	4980	all	uniqgene	uniqtag200	200
all.uniqgene.uniqtag200	65	75	1541	20012	3381	all	uniqgene	uniqtag200	200
all.uniqgene.uniqtag200	70	75	790	22497	896	all	uniqgene	uniqtag200	200
all.uniqgene.uniqtag200	74	75	134	23259	134	all	uniqgene	uniqtag200	200
all.uniqgene.uniqtag200	76	75	2271	20198	3195	all	uniqgene	uniqtag200	200
